# A simple free flap strategy using end-to-side anastomosis to the main vessels in injured extremity

**DOI:** 10.1016/j.jpra.2023.08.003

**Published:** 2023-08-18

**Authors:** Makoto Motomiya, Naoya Watanabe, Mitsutoshi Ota, Kohei Shimoda, Daisuke Kawamura, Norimasa Iwasaki

**Affiliations:** a Department of Orthopaedic Surgery, Obihiro Kosei Hospital Hand Centre, Obihiro, Japan; b Department of Orthopaedic Surgery, Higashisaitama General Hospital, Satte, Japan; c Department of Orthopaedic Surgery, Faculty of Medicine and Graduate School of Medicine, Hokkaido University, Sapporo, Japan; d Department of Orthopaedic Surgery, NTT East Japan Sapporo Hospital, Sapporo, Japan

**Keywords:** End-to-side anastomosis, Extremities soft tissue defect, Free flap, Recipient vessel, Main artery, Strategy

## Abstract

**Background:**

During free flap surgery, the surgeon sometimes encounters problems with anastomosis such as intractable arterial spasms or vessel size discrepancy in venous anastomoses. End-to-side (ETS) anastomosis has the advantages of limited chance of vessel spasm and easy handling by adjusting for vessel size discrepancy. We introduced the arterial and venous end-to-side anastomosis (AV-ETS) strategy, which is based on the ETS anastomosis to the main artery and accompanying veins, to avoid intraoperative anastomotic problems when creating a free flap. The aim of this study was to compare flap outcomes and intraoperative anastomotic problems before and after introduction of the AV-ETS strategy in extremity free flap surgery.

**Materials and methods:**

We retrospectively examined 72 consecutive extremity free flaps. Before introducing the AV-ETS strategy, we used the conventional strategy in which the recipient artery was selected according to the number of the remaining main artery and the anastomosis technique was flexibly changed, although the end-to-end (ETE) technique was used in most cases.

**Results:**

The conventional group had 18 flaps and the AV-ETS group had 54 flaps. The rate of flap survival did not differ between these groups, and there were no cases of flap failure after the introduction of the AV-ETS strategy. The AV-ETS group had significantly fewer flaps that required a change in preoperative planning for the recipient artery or anastomotic site of the artery.

**Conclusions:**

The AV-ETS strategy may facilitate reliable preoperative planning and the performance of stable free flap surgery without requiring a flexible response during surgery.

## Introduction

Free flaps are considered an essential procedure for treating extremity soft tissue defects caused by severe trauma or infection.^1^^,^^2^ Careful preoperative planning, including the selection of the recipient vessels, is important to the success of free flap surgery and for avoidance of critical complications.^3^, ^4^, ^5^ When operating on soft tissue defects caused by severe trauma or infection, the serious problem of intractable arterial spasm related to the “zone of injury” may occur more often in the extremities than in the trunk.^3^^,^^6^, ^7^, ^8^, ^9^ In such cases involving an arterial anastomosis in an extremity free flap, it may not be possible to determine whether the recipient artery selected in the preoperative plan can be used safely without intraoperative direct confirmation. In cases of intractable arterial spasm during surgery, a flexible response is sometimes required to be able to choose another recipient artery or appropriate anastomotic site more proximal to the injured vessel using a vein graft.

Vessel size discrepancy between the flap and recipient veins is also a risk for vascular occlusion.^10^^,^^11^ When performing a venous anastomosis, the surgeon must choose a recipient vein with the optimal diameter under intraoperative direct confirmation and may need to take a flexible approach if there is any vessel size discrepancy. However, preoperative planning may be difficult before direct confirmation of the recipient vessels, and a flexible intraoperative response is often required. In such cases, the surgeon's experience is the most important factor for obtaining a stable outcome for free flaps.^3^^,^^4^^,^^12^

For reconstruction of soft tissue defects in the extremities, the conventional strategy is to first select the proximal stump of the injured artery as the recipient vessel and then perform the anastomosis using the end-to-end (ETE) technique.^5^^,^^13^^,^^14^ However, this conventional strategy may mean that damaged vessels within the zone of injury are selected as the recipient vessels; if this occurs, it may become necessary to change the preoperative plan because of intraoperative intractable vasospasm. In addition, venous anastomosis may be difficult in cases with a large vessel size discrepancy between the flap and recipient veins.

Selection of the main artery and accompanying vein with good blood flow as the recipient vessels using the end-to-side (ETS) technique has been recently reported as useful for free flaps in the extremities.^15^, ^16^, ^17^ The advantages of ETS anastomosis include not only ease of coping with vessel size discrepancy but also preservation of the peripheral circulation, which leads to the prevention of spasm in arteries and the avoidance of congestion and occlusion in veins.^6^^,^^18^, ^19^, ^20^, ^21^, ^22^, ^23^ In the arterial and venous end-to-side anastomosis (AV-ETS) strategy, which is based on the ETS anastomosis to the main artery and accompanying veins, the surgeon could select the recipient vessels regardless of the number of remaining main arteries and could always create uniform anastomoses regardless of any discrepancies in the vessel size. This strategy is considered to have advantages, but we have seen no report related to this strategy focusing on the easier preoperative planning and less need for intraoperative flexible response compared with the conventional strategy.

The aim of this study was to compare flap outcomes and intraoperative anastomotic problems before and after introduction of the AV-ETS strategy in free flap surgery in the extremities.

## Patients and methods

This study was approved by our institutional ethics committee (2022-14). Of the 94 free flaps of 76 patients who received free flap surgery in an extremity, for soft tissue defects caused by severe trauma or infection at our centre between April 2015 and March 2022, we retrospectively examined 72 flaps in 59 consecutive patients, after excluding four flaps for toe defects and 18 flaps for finger defects because of no nearby greater vessels in those areas. For each flap, patient and flap demographics, anastomosis details, flap outcomes, and complications were examined. Any intraoperative anastomotic problems related to intraoperative arterial spasm or a vessel size discrepancy ≥1 mm were also examined.^10^ For cases involving two venous anastomoses, only those that met the criteria for both veins were counted. Intraoperative changes in the recipient artery or anastomotic site that required a vein graft were confirmed by checking the preoperative plan and surgical records. Postoperative complications were evaluated using the Clavien-Dindo classification.^24^

### Introduction of the AV-ETS Strategy

All surgeries were performed by four surgeons under the supervision of M.M., an orthopaedic surgeon, after careful preoperative planning, which included the selection of the recipient vessels and methods for performing the anastomoses.

From April 2015 to July 2017, free flap surgery was performed based on the conventional strategy;^14^ that is, when there were multiple remaining main arteries, one of the main arteries was selected as the recipient artery, and the arterial anastomosis was performed using the ETE or flow-through technique. When there was only one remaining main artery in the extremity, the proximal stump of the injured vessel was first selected as the recipient vessel, and the ETE anastomosis was performed. When use of the injured vessel was difficult, vascular anastomosis was performed using the flow-through or ETS technique to the remaining main artery. When a branch of the main artery with good blood flow was found during surgery, the branch was selected as the recipient vessel and the anastomosis was performed using the ETE technique. For venous anastomosis, we searched for optimal veins with small vessel size discrepancy around the recipient artery and performed venous anastomosis using the ETE technique.

**Figure 1:**
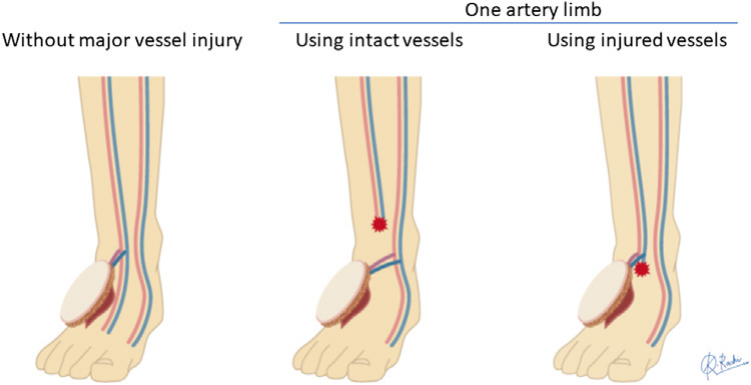
A diagram showing the AV-ETS strategy. The main artery and accompanying veins that travel closest to the soft tissue defect and had good blood flow were selected as the recipient vessels, and all anastomoses were performed using the ETS technique regardless of the number of remaining main arteries. AV-ETS, arterial and venous end-to-side anastomosis; ETS, end-to-side.

**Figure 2:**
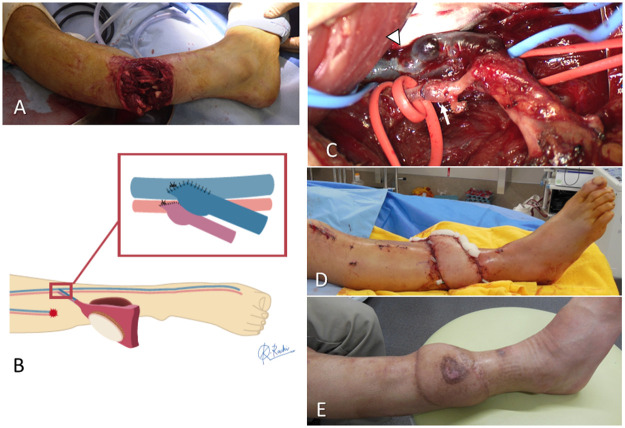
A representative case showing the use of intact vessels as the recipient vessels in the AV-ETS group. **A**: A 35-year-old man was stabbed by the blade of a mowing machine and experienced an open tibial shaft fracture with crushed muscle in the right lower leg. **B**: Diagram showing the free flap surgery using the AV-ETS strategy. Soft tissue reconstruction using a latissimus dorsi flap and internal fixation using an intramedullary nail were performed 4 days after the injury. **C**: The intact tibialis posterior artery and the accompanying vein were selected as the recipient vessels, and arterial anastomosis (arrow) and venous anastomosis (arrowhead) were performed using the MPETS technique. **D, E**: The postoperative finding just after and 13 months after free flap surgery. The flap survived without any postoperative problems, and good function was obtained after tendon reconstruction surgery. AV-ETS, arterial and venous end-to-side anastomosis; MPETS, microscopic parachute ETS.

**Figure 3:**
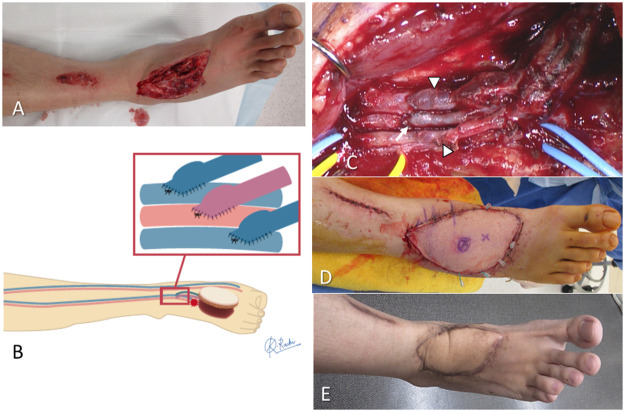
A representative case showing the use of the injured vessels as the recipient vessels in the AV-ETS group. **A**: A 31-year-old man experienced an open fracture dislocation of the right Lisfranc joint after falling from a high place. After debridement and internal fixation surgery, soft tissue reconstruction was performed using an anterolateral thigh flap 10 days after the injury. **B**: Diagram showing the free flap surgery using the AV-ETS strategy. **C**: A site with sufficient blood flow proximal to the injured stump of the dorsalis pedis artery was selected as the recipient vessel and an arterial anastomosis (arrow) and two venous anastomoses (arrowheads) were performed using the MPETS technique. **D, E**: The postoperative finding just after and 1 year after free flap surgery. The flap survived completely without any postoperative problems, and good contour was obtained after defatting procedure. AV-ETS, arterial and venous end-to-side anastomosis; MPETS, microscopic parachute ETS.

The AV-ETS strategy for free flaps was introduced in August 2017 (Figure 1). Regardless of the number of remaining main arteries, the main artery and accompanying veins that travelled closest to the soft tissue defect and had good blood flow to the periphery were selected as the recipient vessels (Figure 2). If the injured main vessel was used, the end of the injured stump was never exposed, and the proximal site of the injured vessel with good blood flow far from the injured stump was selected as the anastomotic site of the recipient vessels (Figure 3). The superficial vein was selected as the recipient vein only if the accompanying vein was very thin and the condition of the superficial vein running near the recipient artery was good.

We always used the microscopic parachute ETS (MPETS) technique as the anastomosis technique. This technique included a wide-slit vesselotomy of the recipient vessel and a parachute technique at the heel of the flap vessel, where anastomosis can be difficult, for both arterial and venous anastomosis.^25^^,^^26^ For the postoperative regimen, prostaglandin E1 was administered continuously at 80 μg/day until postoperative day 4, then at 40 μg/day until day 7, except for patients with comorbidities such as liver dysfunction. Bed rest was ordered for about 1 week, and smoking was prohibited for at least 3 weeks after surgery.

### Statistical Analysis

The patient and flap demographics, anastomosis details, intraoperative problems, and flap outcomes were compared between the conventional and AV-ETS groups using Fisher's exact test, chi-square test, or Welch's *t*-test. A *p* value <0.05 was considered to be significant. All analyses were performed using Bell Curve for Excel (version 3.21, Social Survey Research Information Co., Ltd., Osaka, Japan).

## Results

### Patient and Flap Demographics

The patients were 43 men and 16 women, with an average age of 56 years (range 22–87 years). The average follow-up duration for each flap was 23 months (2–84 months). In two patients, the flap surgery was performed twice during the periods before and after introduction of the AV-ETS strategy. Excluding these two, 13 patients were in the conventional group and 44 in the AV-ETS group. Except for sex distribution, the patient background characteristics did not differ significantly between groups (Table 1). The conventional group had a total of 18 flaps and the AV-ETS group had a total of 54 flaps. The locations of soft tissue defects were 31 in an upper limb and 41 in a lower limb. The indications for free flap surgery were 42 for trauma and 25 for infections. The types of flaps were 52 fasciocutaneous flaps and 14 musculocutaneous flaps. The characteristics of the free flaps did not differ significantly between the two groups (Table 2).

**Table 1:** Patient demographics.

	Total	Conventional	AV-ETS	*p*
Total number of patients	59#	13	44	
Total number of flaps	72#	16	52	
Sex				0.01*
Men	43	13	28	
Women	16	0	16	
Mean age (years)	56 ± 17	52 ± 16	58 ± 16	0.24
Comorbidities				
Hypertension	11	6	11	0.18
Diabetes	16	5	16	1.00
Hemodialysis	5	1	6	1.00
Tobacco use	15	7	11	0.09
Anticoagulant	2	1	5	1.00
PVD	2	0	5	0.58

AV-ETS, arterial and venous end-to-side anastomosis; PVD, peripheral vascular disease.

# Two patients belonged to both groups; their first flaps were Conventional and second flaps were AV-ETS.

⁎ *p* < 0.05

**Table 2:** Locations, indications, and flap types.

	Conventional	AV-ETS	*p*
Total number of flaps	18	54	
Locations			0.28
Upper extremity	10	21	
Upper arm	0	1	
Elbow	0	2	
Forearm	3	9	
Hand#	7	9	
Lower extremity	8	33	
Knee	0	2	
Lower leg	6	11	
Foot#	2	20	
Indication			0.38
Trauma	13	29	
Infection	4	21	
Others	1	4	
Flap type			0.55
Fasciocutaneous	14	38	
ALT	14	36	
PIA	0	1	
Scapula	0	1	
Musculocutaneous (all LD)	2	12	
Others	2	4	
VFG	2	2	
TAP+VSG	0	2	

# Fingers and toes were excluded. ALT, anterolateral thigh flap; AV-ETS, arterial and venous end-to-side anastomosis; PIA, posterior interosseous artery flap; LD, latissimus dorsi myocutaneous flap; VFG, vascularised fibular graft; TAP, thoracodorsal artery perforator flap; VSG, vascularised scapular graft.

### Details of the Anastomoses

Details of the vascular anastomoses are shown in Table 3. Main artery injury caused by trauma was observed in five flaps in the conventional group and in 14 flaps in the AV-ETS group. The percentage of flaps that used the injured artery as the recipient artery was significantly higher in the conventional group than in the AV-ETS group. The main artery was selected for all flaps in the AV-ETS group, and the selection of the recipient artery differed significantly between the two groups. Although the number of venous anastomoses did not differ significantly between the two groups, for the type of vein selected, significantly more flaps used the deep veins accompanying the main artery in the AV-ETS group than in the conventional group. Regarding the arterial anastomotic type, in the conventional group, different anastomosis methods such as ETE, flow-through, and ETS were selected for each case, but in the AV-ETS group, almost all cases received ETS treatment. In addition, venous anastomosis was ETE in almost all cases in the conventional group, while ETS was used in almost all cases in the AV-ETS group. In the first two patients in the AV-ETS group, one of the two venous anastomoses was anastomosed using ETS and the other using ETE.

**Table 3:** Anastomosis details.

	Conventional	AV-ETS	*p*
Total number of flaps	18	54	
Main artery injury	5 (28%)	14 (26%)	0.76
successfully repaired	0	5	
Recipient artery			0.003*
Great artery	14 (78%)	54 (100%)	
Brachial artery	0	5	
Radial artery	5	16	
Ulnar artery	2	0	
Femoral artery	0	2	
Anterior tibial artery	2	3	
Posterior tibial artery	4	11	
Dorsal pedis artery	1	17	
Branch of a great artery	4 (22%)	0 (0%)	
Upper extremity	3	0	
Lower extremity	1	0	
Using an injured great artery or its branch as recipient	4 (80%)	3 (21%)	0.04*^,^$
Arterial anastomosis type			<0.001⁎⁎
ETE	9 (50%)	1# (2%)	
Flow-through	5 (28%)	0 (0%)	
ETS	4 (22%)	53 (98%)	
Recipient vein			
Number of anastomotic veins			0.59
2	8 (44%)	29 (54%)	
1	10 (56%)	25 (46%)	
Types of veins			<0.001**
Deep vein(s) only	4 (22%)	46 (85%)	
Superficial vein(s) only	8 (44%)	5 (9%)	
Deep vein and superficial vein	6 (33%)	3 (6%)	
Venous anastomosis type			<0.001**
ETE	25 (96%)	2 (2%)	
ETS	1 (4%)	81 (98%)	

AV-ETS, arterial and venous end-to-side anastomosis; ETE, end-to-end anastomosis; ETS, end-to-side anastomosis.

# One arterial anastomosis was performed using an ETE because of failure of ETS.

$ Rate of using an injured artery or its branch in a flap with a main artery injury.

⁎ *p* < 0.05 ** *p* < 0.001

### Intraoperative Problems

Intraoperative anastomotic problems such as intractable arterial spasms occurred in three patients in the conventional group but in no patients in the AV-ETS group. Vessel size discrepancy ≥1 mm occurred in 33 flaps for arterial anastomosis and in 20 flaps for venous anastomosis; the vessel size discrepancy did not differ significantly between the two groups. In the conventional group, the recipient artery was changed to a more suitable artery for three flaps, and the anastomotic site was changed to the proximal site using a vein graft for two flaps because of intraoperative arterial spasm. In the AV-ETS group, the recipient artery was changed for only one flap because the route of the flap pedicle vessels was likely to be squeezed. In the AV-ETS group, the anastomotic site was changed to the proximal site for only one flap because the recipient artery was damaged by excessive traction during recipient vessel preparation, and an ETE anastomosis was performed using a vein graft. Significantly fewer flaps required a change in the recipient artery or anastomotic site in the AV-ETS group than in the conventional group (Table 4).

**Table 4:** Intra-operative problems.

	Conventional	AV-ETS	*p*
Total number of flaps	18	54	
Intra-operative artery spasm	3 (17%)	0 (0%)	0.01*
Artery size discrepancy ≥1 mm	6 (33%)	27 (50%)	0.28
Vein size discrepancy ≥1 mm ^#^	5 (28%)	15 (28%)	1.00
Change in the arterial recipient	5 (28%)	2 (4%)	0.009⁎⁎
Change in the recipient vessel	3	1	
Change in the anastomotic site using a vein graft	2	1	

AV-ETS, arterial and venous end-to-side anastomosis.

^#^For flaps with two anastomosed veins, only those with both vein size discrepancies ≥1 mm were included.

⁎ *p* < 0.05

⁎⁎ *p* < 0.01

### Flap Outcomes and Complications

Seventy of the 72 flaps survived (Table 5). In the severe postoperative complications, most of the grade IIIa and IIIb complications were flap-related, while four cases with grade IV complications had serious systemic issues. In the AV-ETS group, one case required intensive care unit management due to postoperative blood pressure instability, one case had acute myocardial infarction, and one case had pneumonia. All cases were finally cured without sequelae. Two flaps exhibited complete loss in the conventional group because of intractable arterial spasm. An attempt at reanastomosis was abandoned because a marked white thrombus was found in the flap artery in these two flaps. Because of excessive defatting procedures or flap harvest beyond the angiosome, five flaps exhibited partial necrosis but obtained complete healing after a skin graft and secondary closure. Arterial occlusion occurred in one flap in the AV-ETS group in which the arterial anastomosis was changed to the ETE technique because of iatrogenic injury of the recipient artery, but the flap survived after reanastomosis. Of the two flaps that exhibited venous congestion, one had a short pedicle vessel length, which led to pedicle kinking during extension of the adjacent joint. The other exhibited venous pedicle kinking caused by insufficient separation of the artery and veins in the flap pedicle. Both of these flaps survived after venous reanastomosis. Postoperative deep infection was found in 16 flaps, and debridement under the flap was performed early after free flap surgery on 19 flaps based on the concept of the “close-open-close technique”.^27^ The flap outcomes and complications did not differ significantly between the two groups.

**Table 5:** Flap outcomes and complications.

		Conventional	AV-ETS	*p*
Total number of flaps		18	54	
Number of flaps that survived		16 (89%)	54 (100%)	0.06
Postoperative complication$	No complication	5	24	0.75
	Grade I	3	7	
	Grade II	0	0	
	Grade IIIa	4	11	
	Grade IIIb	5	9	
	Grade IV	1	3	
Flap-related complication		8 (44%)	20 (37%)	0.59
Complete flap loss		2 (11%)	0 (0%)	0.06
Partial flap necrosis		2 (11%)	3 (6%)	0.59
Arterial occlusion		2 (11%)	1# (2%)	0.15
Venous occlusion		0 (0%)	2 (4%)	1.00
Deep wound infection		2 (11%)	13 (24%)	0.33
Wound dehiscence		1 (6%)	3 (6%)	1.00
Delayed osteomyelitis		2 (11%)	1 (2%)	0.15
Additional surgery		16 (89%)	38 (70%)	0.21
Take-back revision anastomosis		0 (0%)	3 (6%)	0.57
Secondary free flap		2 (11%)	0 (0%)	0.06
Debridement under the flap soon after flap surgery		4 (22%)	15 (28%)	0.76
Debridement for delayed osteomyelitis		2 (11%)	1 (2%)	0.15
Additional functional reconstruction		8 (44%)	16 (30%)	0.26
Skin graft and/or secondary closure		5 (28%)	7 (13%)	0.16
Defatting		5 (28%)	8 (15%)	0.29

AV-ETS, arterial and venous end-to-side anastomosis.

$ According to the Clavien–Dindo classification^24^.

# One arterial occlusion occurred in a case of ETE arterial anastomosis in the AV-ETS group.

## Discussion

We introduced the AV-ETS strategy when using free flaps to treat soft tissue defects in the extremities caused by trauma and infection (Figure 1). Unlike the conventional strategy, in the AV-ETS strategy, the main arteries and accompanying veins closest to the soft tissue defect, which had good blood flow, were selected as the recipient vessels regardless of the number of remaining main arteries, and all anastomoses were performed using the ETS technique. Although the flap survival rate did not differ significantly from before to after introduction of the AV-ETS strategy, we found no flap failure after its introduction. In addition, after introduction of the AV-ETS strategy, significantly fewer flaps required a change in preoperative planning for the recipient artery or arterial anastomotic site. Although it is not clear whether the ETE or ETS is suitable for both arterial and venous anastomoses,^28^, ^29^, ^30^, ^31^ the AV-ETS strategy may make it possible to develop a reliable preoperative plan to create a stable free flap without requiring a flexible response during surgery.

Because a completely transected artery tends to retract, contract, and stop bleeding,^6^^,^^19^ intraoperative intractable spasm is likely to occur to the damaged artery within the zone of injury in the ETE anastomosis, even if the preoperative examination shows it has good blood flow.^3^^,^^8^^,^^9^ In our study, three flaps in the conventional group that had used the stump of the injured artery as the recipient artery exhibited intractable arterial spasm, which was followed by complete flap failure in two flaps. Intractable vessel spasm is less likely to occur in the recipient artery in the ETS anastomosis because the vessel continuity is not compromise.^6^^,^^15^^,^^16^ No intraoperative spasm was encountered during anastomosis after introduction of the AV-ETS strategy. Although the use of the only remaining main artery tends to be avoided in a limb with one artery,^13^^,^^14^ this procedure may be appropriate if performed properly given that previous studies have reported good patency in the recipient main artery after the ETS anastomosis.^26^^,^^32^

Selection of the recipient vein and proper venous anastomosis are also important for avoiding serious complications in free flaps.^8^^,^^10^^,^^33^ In the selection of recipient veins, superficial veins are thick and firm but may be damaged by trauma and infection, whereas the deep veins that accompany the main artery are less susceptible to damage and may be more reliable as recipient veins.^4^^,^^34^^,^^35^ However, deep veins are likely to have a smaller diameter than flap veins, and venous anastomosis with a vessel size discrepancy is often difficult using the ETE technique.^21^^,^^36^ Although venous ETS is used less frequently in the extremity than in the head and neck,^21^^,^^37^ venous ETS has various advantages including easy handling of a vessel size discrepancy, easy adjustment of the anastomotic site, and avoidance of venous congestion because of the pump effect of peripheral tissues.^10^^,^^16^ In most cases in our study, the deep vein was selected as the recipient vein after introduction of the AV-ETS strategy, and venous anastomosis with >1 mm vessel size discrepancy was easily performed using the venous ETS technique in 15 flaps. Because the surgeon can select the accompanying vein as the recipient vein without seeking another suitable vein when using the venous ETS technique, we believe that the AV-ETS strategy will make soft tissue reconstruction less invasive.^35^^,^^38^

A stable ETS technique is required when introducing the AV-ETS strategy. ETS is a more complicated procedure than ETE, and no single method has been shown to be superior, although various ETS techniques have been reported.^16^^,^^22^ We have previously reported the MPETS technique as a simple and reliable method for ETS anastomosis.^25^^,^^26^^,^^38^ The MPETS method has many advantages, including a simple slit-shaped vesselotomy, large window to avoid stenosis, and reliable sutures using the parachute technique in the heel of the flap vessels where blood leakage is likely to occur. The MPETS technique can be applied to large and small vessels, as well as arteries and veins by performing the procedure under a microscope. This technique is easy to teach to other surgeons and can be learned in off-the-job training. Although minor modifications are sometimes needed in cases with arteriosclerotic vessels, we believe that this technique is a useful ETS procedure for free flap anastomosis.

The surgeon's experience is considered to be the most important factor in determining the outcome of the free flap technique.^3^^,^^4^^,^^12^ However, in these days when the free flap technique has been established, it is not acceptable to repeat the failure of free flaps even by an inexperienced team. The usefulness of constructing a good educational system for disseminating microsurgical skills has been reported previously.^39^^,^^40^ We believe that the AV-ETS strategy using the MPETS method ensures that preoperative planning and intraoperative procedures are uniform. Hence, the MPETS method is useful for passing on the free flap technique. The current ETS technique is time consuming compared with ETE.^25^ Recently, coupler devices have been widely used for venous anastomosis of free flaps, and there are many reports on their usefulness.^41^, ^42^, ^43^ By incorporating the venous coupler into the AV-ETS strategy, there is a possibility that extremity free flap surgery can be performed more easily, reliably, and in a short time. We plan to verify this strategy in the future.

We believe that surgeons should consider the following three items to ensure the safe use of the AV-ETS strategy with the MPETS technique.

1) As previously described by Godina,^6^ it is important to collect sufficient length of the flap pedicle so that it can reach the planned anastomotic site.
2) Because of the large vesselotomy to the recipient vessel, exposure of the recipient vessels in the MPETS technique should be larger than when performing the common ETS with a small vesselotomy.
3) The artery and veins of the flap pedicles need to be well separated to avoid becoming kinked because all vessels will be anastomosed to the adjacent vessels.

Although vascular occlusion occurred in three cases in the AV-ETS group, we believe that these complications could have been avoided by considering these precautions. In the unlikely event that the recipient artery is damaged by the intraoperative procedure, it would be better to reconstruct the injured main artery first using a vein graft and then perform the ETS again using the implanted vein as the recipient artery.

In this study, many serious postoperative complications were observed in addition to blood flow disorders in the free flap. Due to the relatively large number of infected cases, many deep infections were observed as postoperative complications. We should have performed an appropriate culture test and confirmed that the bacteria had been removed after debridement before performing the free flap technique.^44^ In addition, serious postoperative systemic complications were observed in patients with comorbidities. Since prolonged general anaesthesia increases perioperative complications,^45^ we are considering the introduction of a free flap technique that is safe for systemic hemodynamics using spinal/epidural anaesthesia without general anaesthesia.^46^^,^^47^ In performing a stable free flap technique, it is necessary to consider not only the technical acquisition of the free flap but also the reduction of complications.^48^

Our study has some limitations. First, this was a retrospective study with a small number of flaps, and the patient and flap backgrounds did not match perfectly in the two groups. Second, due to the different sample sizes between the two groups, statistically significant difference may not have been adequately detected. Although the number of cases in the conventional group cannot be increased because of the introduction of the AV-ETS strategy, studies with larger sample sizes may show significant differences in clinical outcomes between the two groups. Finally, the difference in treatment time between the conventional and AV-ETS groups means that the operator's learning curve may have been an uncontrolled factor in this study. Especially in the early cases of the conventional group, there was a tendency to choose the proximal stump of the damaged vessel and to use the ETE technique rather than the unfamiliar ETS, which is thought to sometimes result in arterial spasm.

## Conclusions

In this study, we compared the flap outcomes and intraoperative anastomotic problems for extremity free flap surgery before and after introduction of the AV-ETS strategy. Unlike the conventional strategy, in the AV-ETS strategy, the main arteries and accompanying veins closest to the soft tissue defect, which had good blood flow, were selected as the recipient vessels regardless of the number of remaining main arteries, and all anastomoses were performed using the ETS technique. The rate of flap survival did not differ significantly between the groups, but there were no cases of flap failure after introduction of the AV-ETS strategy. In addition, significantly fewer flaps required a change in the preoperative planning for the recipient artery or the anastomotic site of the artery after introduction of the AV-ETS strategy. When operating on soft tissue defects caused by severe trauma or infection, the AV-ETS strategy may facilitate reliable preoperative planning and the performance of stable free flap surgery without requiring a flexible response during surgery.

## Statements and Declarations

### Funding

None.

## Conflicts of interest statement

None.
